# Characterization of parameters for the analysis of objective measures of non-nutritive sucking of newborns

**DOI:** 10.1590/2317-1782/20242023149en

**Published:** 2024-05-31

**Authors:** Bárbara Generoso Santos de Matos Sales, Renata Maria Moreira Moraes Furlan, Camila Alexandra Vilaça Ramos, Narciso Sena Fracaroli, Estevam Barbosa de Las Casas, Andréa Rodrigues Motta

**Affiliations:** 1 Programa de Pós-graduação em Ciências Fonoaudiológicas, Universidade Federal de Minas Gerais – UFMG - Belo Horizonte (MG), Brasil.; 2 Departamento de Fonoaudiologia, Faculdade de Medicina, Universidade Federal de Minas Gerais – UFMG - Belo Horizonte (MG), Brasil.; 3 Programa de Pós-graduação em Engenharia Mecânica, Universidade Federal de Minas Gerais – UFMG - Belo Horizonte (MG), Brasil.; 4 Universidade Federal de Minas Gerais – UFMG - Belo Horizonte (MG), Brasil.; 5 Departamento de Engenharia de Estruturas, Escola de Engenharia, Universidade Federal de Minas Gerais – UFMG - Belo Horizonte (MG), Brasil.

**Keywords:** Non-nutritive Sucking, Sucking Behavior, Newborn, Measuring Equipment, Breast-Feeding

## Abstract

**Purpose:**

To propose a methodology for analyzing data generated by an instrument measuring non-nutritive sucking pressure in newborns.

**Methods:**

An analytical observational study was developed, with a cross-sectional design, considering the data collected from 24 full-term newborns without complications. Three collections from each neonate were analyzed, with duration of 2 minutes and a 2-minute interval between them. The defined parameters were extracted using a program developed in Matlab®. The results were obtained by analyzing and comparing 12 variables at a 5% confidence level. Comparison of manual and computerized analyzes was also carried out using the intraclass correlation coefficient.

**Results:**

The multiple comparison between the three collection moments showed that the significant statistical differences occurred between collections one and two and two and three. When analyzing and comparing each variable separately, it was noted that the second collection showed: greater number of sucking groups, greater number of suctions, less time to start the sucking groups, longer time of sucking groups, less number of sporadic suctions, higher mean pressure values and with less standard deviation, more number of pauses with shorter time of pauses. The intraclass correlation coefficient revealed almost perfect agreement for the 12 evaluated parameters.

**Conclusion:**

The 12 variables analyzed are relevant, especially in the second collection. The Matlab® program proved to be viable and effective in extracting and analyzing parameters, showing high agreement when compared to manual evaluation.

## INTRODUCTION

Full-term newborns (NB) without any changes at birth are expected to be able to feed orally, without any compromised vital function. To this end, the functions of sucking, swallowing, and breathing must be coordinated and harmonious, keeping characteristics such as lip sealing, adequate tongue and jaw movements, sucking rhythm, and pauses^(1,2)^.

There are two types of suction, namely: non-nutritive sucking (NNS), in which suction movements are performed without introducing liquid into the oral cavity, and nutritive sucking (NS), in which the baby removes liquid from the breast or container to the oral cavity for feeding^(1)^. Both types share parameters that can be described in terms of suction pattern, rate, rhythm, and pressure, with distinct patterns of consecutive sucking and pauses^(2)^.

NNS integrates a set of skills necessary for the baby to mature and develop^(1)^ and brings many benefits to the NB, such as crying less and having their pain eased when undergoing painful clinical procedures. NNS stimulation helps to adapt the oral muscles and stimulates gastric motor function, facilitating weight gain, which reduces the length of hospital stay^(3)^.

Furthermore, NNS is predictive of the beginning of oral feeding, indicating NS would be possible^(4)^. Factors such as prematurity, neurological changes, and craniofacial malformations can influence NB’s sucking behavior, leading to difficulties with safe feeding, and impairing the development of the baby's stomatognathic functions^(1)^.

Speech-language-hearing pathologists are professionals qualified for NNS assessment and training in Brazil. However, few protocols and scales are currently available for clinical use. Validated qualitative scales like NEIVA^(4)^, Neonatal Oral-Motor Assessment Scale (NOMAS)^(5)^, FUGINAGA^(6)^, and XAVIER^(7)^ are based on descriptive observation of babies' oral motor behavior, also evaluating other aspects of the NB's global development. These scales have in common the categorical classification of their findings, such as present, absent, weak, strong, adequate, inadequate behaviors, and so forth, and their answers may vary according to the evaluator's experience.

Few studies have addressed the use of instruments for the quantitative assessment of suction^(8-15)^, and most of them focus on NS, seldom approaching NNS findings.

Some of these quantitative studies do not report how they analyze the data they have detected and recorded with the measuring equipment. The methods reported include the mother's judgment of sucking behavior associated with quantified patterns^(8)^; the comparison of blind assessments between observers^(9)^; the mention of customized programs or software for data extraction^(10-12)^; and the use of Matlab^®^ software^(13,14)^ for processing biological signals and applying independent evaluative methods for comparison^(15)^.

Thus, the paucity of quantitative studies, sometimes with a quick and superficial data processing approach, contributes to the difficulty of systematizing NNS behavior analysis methods.

Given this need, the Biomechanical Engineering Group at the Federal University of Minas Gerais proposed a measuring system^(16)^ to help clinically assess NNS. However, it is essential to not only develop such an instrument but also define parameters to analyze the data it generates and present a systematized method for using it, thus standardizing and validating the equipment.

Using a quantitative method to assess NNS is a scientific advance, contributing to speech-language-hearing clinical practice, providing the same assessment perspective to all professionals involved in a case, and positively influencing the discussion of therapeutic approaches.

Therefore, this study aimed to propose a method to analyze data generated by an instrument that measures NB’s NNS pressure.

## METHOD

This is a cross-sectional analytical observational study based on data collected for the research by Ramos^(16)^, after approval by the Research Ethics Committee of the institution of origin, under number 32078014.0.0000.5149.

**Figure 1:**
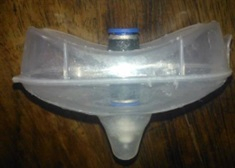
Instrument to measure non-nutritive sucking^(16)^

Source: Ramos^(16)^

The said research^(16)^ developed a method for instrumental NNS assessment (Figure 1) consisting of a probe tip (component introduced into the oral cavity), connectors (connecting the probe tip to the vacuum sensor), a vacuum sensor – CRM-5-10 Sensum (which captures negative pressure and generates a signal treated, transmitted, processed, and stored in digital media), and a data storage and analysis system (NI USB 6008, National Instruments, Austin, TX).

After calibrating the sensor at the Isaac Newton Laboratory of the CITSF Metrology Management – CETEC Campus, a 17% expanded maximum relative measurement uncertainty was obtained at -5 kPa.

The measurements were taken at the Sofia Feldman Hospital – Comprehensive Healthcare Foundation. The research sample^(16)^ comprised 30 full-term babies with a gestational age equal to or greater than 37 weeks; adequate birth weight; intact structures and functions of the oral sensorimotor system; no lingual frenulum changes, craniofacial malformations, or neurological, clinical, or respiratory changes; preserved feeding capacity; and on exclusive breastfeeding, regardless of sex, hours of life, or type of birth. The NBs’ parents/guardians signed an informed consent form.

The babies underwent two assessment methods by the same evaluator, with more than 10 years of experience in the area. They were initially submitted to the qualitative NNS protocol^(4)^ and then to the objective NNS assessment with the instrument in question (Figure 1).

During the two methods carried out in primary research^(16)^, the NBs were assessed on the researcher's lap, in the supine position, supported on the neck, keeping the head and neck higher than the rest of the body. For the qualitative assessment, the researcher's gloved little finger was inserted between the NB's lips and kept in his oral cavity for 2 minutes. Then, for objective assessment, the baby's position was maintained as described above, and the gloved finger was replaced by the instrument’s test tip (Figure 1), which was introduced between the NB's lips to capture the suction pressure.

After the clinical assessment, the researcher took three measures from each NB, lasting 2 minutes each with 2-minute rest intervals between measurements.

The primary research inclusion criteria^(16)^ were NBs with adequate suction pressure in the clinical NNS assessment with the subjective assessment protocol^(4)^ and whose parents/guardians signed the informed consent form. The exclusion criteria^(16)^ were NBs who were continuously crying or irritated during the assessments or did not suck during the measurement.

New exclusion criteria had to be defined for the present study to enable the analysis of records obtained in the research by Ramos^(16)^.

The exclusion criteria were as follows: NBs with one or more recordings with interference (repeated and continuous noises over time), without at least one suction group per collection, that did not reach the time of two minutes per collection, as well as presenting duplication of tracings (pressure and time data replicated at the time the files were generated in the equipment's storage system)^(16)^.

Thus, using the exclusion criteria of the primary research and the present study, the final research sample had 24 NBs – 15 (62.5%) males and nine (37.5%) females –, with a mean age of 19.5 hours, a mean gestational age of 39.1 weeks, a mean birth weight of 3,307.08 grams, and a mean subjective assessment score^(4)^ of 75.50.

A pressure value had to be established before beginning the analysis, indicating which signals could be counted as suctions. This threshold mainly prevents interference and possible movements from being unduly assumed as suctions.

Hence, tests were carried out to determine a value that not only indicated a pressure threshold to count the suctions, but that was also capable of considering each NB’s performance. The tests calculated the mean pressure of the entire trace plus variations in its standard deviation.

The calculation of confidence intervals showed that the mean pressure signal added to the standard deviation could be assumed as the threshold, thus disregarding small interferences and enabling variation according to the NB’s performance at each measurement.

Thus, signals above the threshold – i.e., the mean of all pressure signals plus the standard deviation – were defined as suctions. To differentiate suction events, two very close suctions were counted as independent events if the two pressure variations exceeded the previously established threshold. Otherwise, the two deflections were counted as parts of the same event, and the largest deflection was the peak value^(12)^.

**Figure 2:**
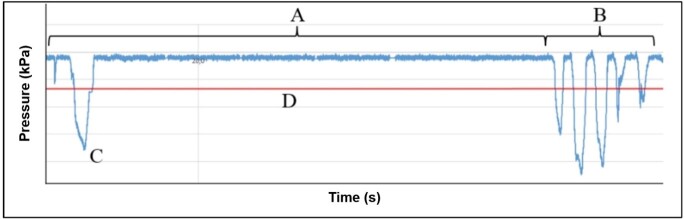
Parameters defined for the study

After determining the pressure threshold, the following parameters were defined considering the parameters investigated in the literature^(2,4,9-12,14,15)^ to systematize the extraction of variables and minimize the possibility of measuring inconsistent values:

1. Number of suction groups: Suction groups are characterized by the presence of three or more suctions with time intervals of less than 3 seconds between suctions^(4)^ (Figure 2).

Caption: A = pause, B = suction group, C = sporadic suction, D = suction threshold
2. Number of suctions: Number of suctions in the tracing that are part of suction groups.
3. Time to start suction groups: Time (s) spent by the NB until the first sucking group begins.
4. Suction group time: Time (s) elapsed in the suction group.
5. Suction frequency (f): Number of suctions performed per second – the opposite of the period (n/s).
6. Suction period: Interval between two consecutive suction peaks in a group (1/f).
7. Number of sporadic suctions: Isolated suction events that are not part of any suction group (Figure 2).
8. Minimum pressure value: Highest suction amplitude of the groups (kPa).
9. Mean pressure value: Mean of pressure peaks (kPa) in the groups.
10. Maximum pressure value: Lowest suction amplitude of the groups (kPa).
11. Number of pauses: Pauses are time intervals greater than or equal to 3 seconds without the presence of a suction group^(4)^ (Figure 2).
12. Pause time: Time (s) spent in pauses.

**Figure 3:**
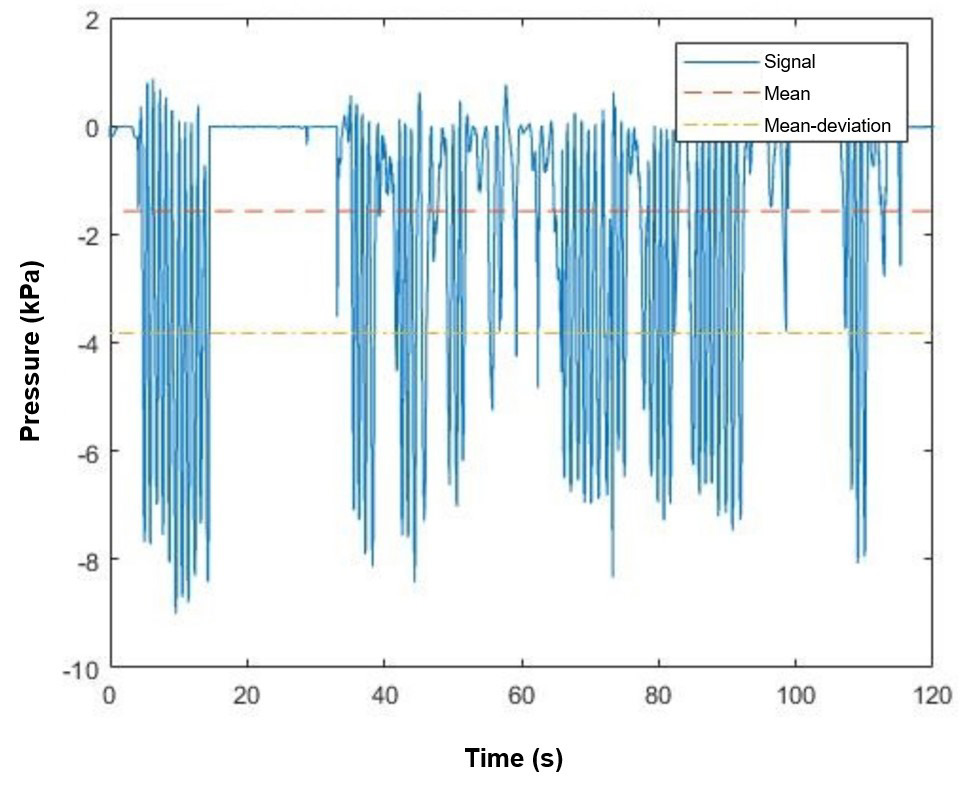
Graph with corrections

**Figure 4:**
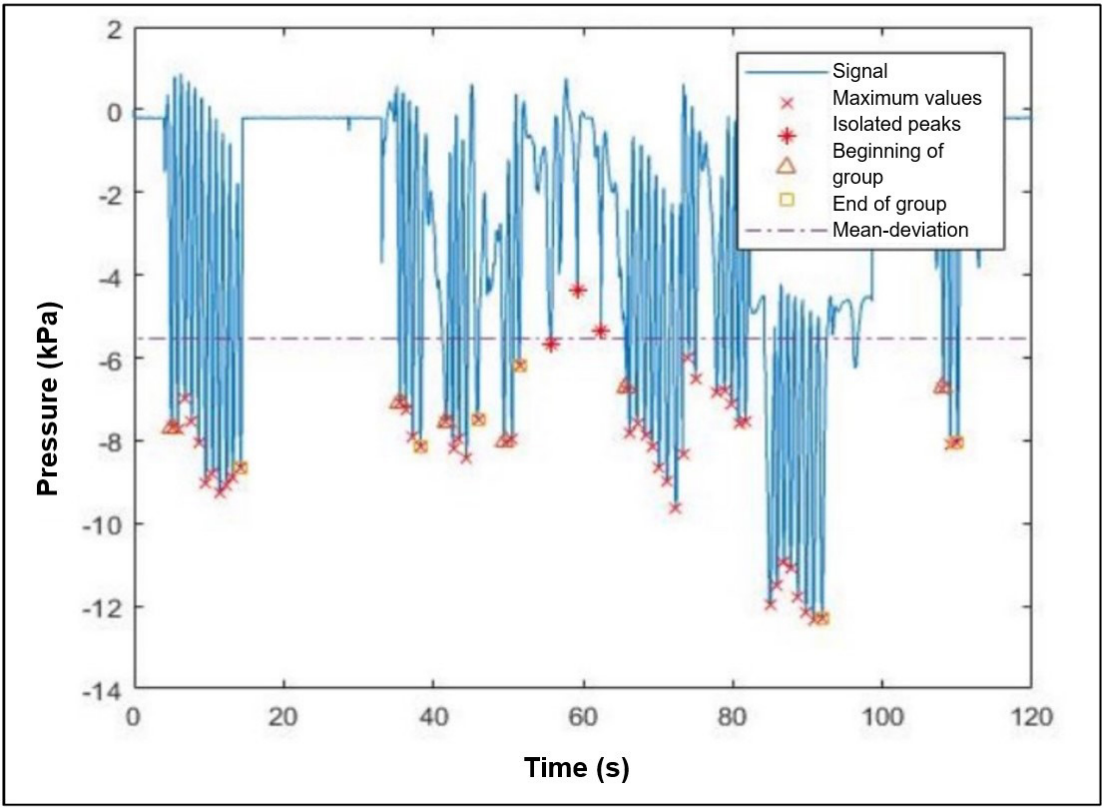
Graph without corrections

The UFMG Biomechanical Engineering Group developed a program in Matlab^®^ to analyze the records. The treatment initially used a low-pass Finite Impulse Response (FIR) filter. An auxiliary curve was created to simplify peak identification, obtained by correcting the original curve (Figure 3). Then, the researchers applied the treatment based on a proposed method^(12)^, with the necessary adaptations, to filter out non-relevant points initially identified. Thus, with all known maximum values in the original curve (Figure 4), the groups were recognized, and operations were made to determine the variables of interest.

After programming the software, the extracted variables were transcribed and organized in an Excel database.

Six NBs (25% of the total sample) were randomly selected among those that allowed manual analysis for subsequent agreement analysis, verifying the agreement between manual and computerized analyses.

The variables of interest were characterized by measures of central tendency and dispersion. Data were analyzed in SPSS software, version 21.0, using the non-parametric Friedman and Wilcoxon tests at 5% significance to compare the values of each variable between the three collections. The intraclass correlation coefficient (ICC) verified the agreement between the analyses with two different evaluation methods.

## RESULTS

The descriptive analysis of topics from the NNS Assessment Form^(4)^ revealed that most NBs were born through normal birth (75.0%), started sucking easily (70.8%), always had strong (70.8%) and rhythmic (66.7%) sucking, and never showed signs of stress (83.3%) during the assessment.

Table 1 shows the measures of central tendency and dispersion of variables related to suction events, namely: the number of suction groups, number of suctions, time to start suction groups, suction group time, suction frequency, suction period, and number of sporadic suctions.

**Table 1:** Measures of central tendency and dispersion of variables of interest related to suction events

	No. of suction groups	No. of suctions	Time (s) to begin suction groups	Time (s) of suction groups	Suction frequency (n/s)	Suction period (s)	No. of sporadic suctions
Collection 1							
Mean	4.21	29.63	28.60	36.48	0.84	1.29	4.46
SD	2.04	19.37	25.77	22.44	0.17	0.32	2.73
Minimum	1.00	5.00	1.30	4.40	0.53	0.88	0.00
Median	4.00	27.50	17.00	34.20	0.90	1.15	5.00
Maximum	8.00	66.00	93.00	74.50	1.14	1.97	10.00
Collection 2							
Mean	5.21	40.45	16.33	48.73	0.85	1.24	3.04
SD	1.74	18.91	12.39	20.94	0.10	0.19	2.42
Minimum	2.00	11.00	0.90	13.50	0.59	1.01	0.00
Median	6.00	40.00	13.05	52.10	0.84	1.22	2.00
Maximum	8.00	73.00	44.00	84.70	1.02	1.88	9.00
Collection 3							
Mean	4.50	33.25	22.68	39.79	0.90	1.18	3.54
SD	1.74	19.63	22.07	21.06	0.14	0.21	3.50
Minimum	1.00	8.00	0.70	11.10	0.57	0.89	0.00
Median	5.00	33.50	14.30	36.60	0.89	1.14	2.50
Maximum	7.00	77.00	92.10	84.20	1.14	1.76	13.00

Caption: No. = number; SD = standard deviation; s = seconds; (n/s) = number of suctions per second

Table 2 presents measures of central tendency and dispersion of variables related to pressure and pause: minimum pressure, mean pressure, maximum pressure, number of pauses, and pause time.

**Table 2:** Measures of central tendency and dispersion of variables of interest related to pressure in kPa and pause

	**Minimum pressure**	**Mean pressure**	**Maximum pressure**	**No. of pauses**	**Time of pauses (s)**
**Collection 1**					
Mean	- 8.43	- 7.04	- 5.25	4.50	83.52
SD	4.08	3.71	3.29	1.77	22.44
Minimum	- 17.46	- 15.07	- 13.48	2.00	45.50
Median	- 9.21	- 6.69	- 4.56	5.00	85.80
Maximum	- 2.95	- 2.18	- 1.73	8.00	115.60
**Collection 2**					
Mean	- 9.82	- 8.49	- 6.36	5.46	71.28
SD	4.15	3.65	3.39	1.53	20.94
Minimum	- 15.93	- 13.82	- 11.97	2.00	35.30
Median	- 11.20	- 9.43	- 6.24	5.00	67.90
Maximum	- 3.31	- 2.89	- 1.92	8.00	106.50
**Collection 3**					
Mean	- 10.06	- 8.47	- 6.24	4.83	80.21
SD	4.79	4.10	3.77	1.52	21.06
Minimum	- 18.62	- 15.25	- 13.78	2.00	35.80
Median	- 10.69	- 9.16	- 6.77	5.00	83.40
Maximum	- 2.42	- 2.19	- 1.39	8.00	108.90

Caption: No. = number; SD = standard deviation; s = seconds

Table 3 presents the comparison of each variable between the three collections with the Friedman test, revealing statistically significant associations. The table also presents the Wilcoxon test that identifies in which pairs the differences occurred.

**Table 3:** Collections compared two by two

**Variables**	**p-value****1**	**Multiple comparisons** **2** **between collections**		
		**1 x 2**	**2 x 3**	**3 x 1**
No. of suction groups	0.057	-	-	-
No. of suctions	0.005*	0.007*	0.052	1.000
Time to start suction groups	0.115	-	-	-
Suction group time	0.005*	0.018*	0.012*	1.000
Suction frequency	0.034*	0.582	0.028*	0.582
Suction period	0.093	-	-	-
No. of sporadic suctions	0.213	-	-	-
Minimum pressure	0.100	-	-	-
Mean pressure	0.001*	0.001*	0.337	0.130
Maximum pressure	0.331	-	-	-
No. of pauses	0.017*	0.028*	0.250	1.000
Time of pauses	0.005*	0.018*	0.012*	1.000

1 Friedman test;

2 Wilcoxon test;

* p ≤ 0.05

Caption: No. = number

In Table 4 presents the agreement analysis with ICC between the 12 variables of interest, extracted with the two evaluation methods (manual and computerized. The analysis indicated almost perfect agreement for all variables.

**Table 4:** Agreement analysis between assessments of the 12 variables of interest

**Variables**		**Manual analysis**											
		**1^st^**	**2^nd^**	**3^rd^**	**4^th^**	**5^th^**	**6^th^**	**7^th^**	**8^th^**	**9^th^**	**10^th^**	**11^th^**	**12^th^**
Software analysis	1^st^	0.986	-	-	-	-	-	-	-	-	-	-	-
	2^nd^	-	0.997	-	-	-	-	-	-	-	-	-	-
	3^rd^	-	-	0.995	-	-	-	-	-	-	-	-	-
	4^th^	-	-	-	0.967	-	-	-	-	-	-	-	-
	5^th^	-	-	-	-	0.934	-	-	-	-	-	-	-
	6^th^	-	-	-	-	-	0.954	-	-	-	-	-	-
	7^th^	-	-	-	-	-	-	0.900	-	-	-	-	-
	8^th^	-	-	-	-	-	-	-	0.999	-	-	-	-
	9^th^	-	-	-	-	-	-	-	-	0.997	-	-	-
	10^th^	-	-	-	-	-	-	-	-	-	0.995	-	-
	11^th^	-	-	-	-	-	-	-	-	-	-	0.964	-
	12^th^	-	-	-	-	-	-	-	-	-	-	-	0.968

Intraclass correlation coefficient

Caption: 1^st^: Number of suction groups; 2^nd^: Number of suctions; 3^rd^: Time to start suctions groups; 4^th^: Suction group time; 5^th^: Suction frequency; 6^th^: Suction period; 7^th^: Number of sporadic suctions; 8^th^: Minimum pressure; 9^th^: Mean pressure; 10^th^: maximum pressure; 11^th^: Number of pauses; 12^th^: Time of pauses

## DISCUSSION

This study was based on data from primary research to develop an instrument to assess NNS in NBs^(16)^ carried out by the UFMG Biomechanical Engineering Group. The variables found in the previous subjective assessment and the three collections of NNS quantitative assessment of 24 newborns (15 boys and nine girls) were compared to propose a form of record analysis.

### Characterization of parameters

As indicated in the literature, suction behavior can be described in the NB’s suction patterns and rates, suction and pause rhythm, and pressure^(2)^.

Events such as the number, duration, and frequency of suctions, groups of suctions, pauses, pressure, and sucking/swallowing/breathing coordination are monitored simultaneously in routine clinical assessments^(9)^. Therefore, it is essential to objectively measure and review these data^(9)^.

Thus, the parameters in this study were defined based on behaviors investigated in validated qualitative scales and quantitative NS and NNS research. The importance of taking objective measures, enabling subsequent record analysis and standardization of an evaluation method, justifies the choice of these parameters and their inclusion in the computational data analysis performed in this research.

#### Suction

An objective study on NS development patterns^(10)^ established a -16 mmHg threshold for detecting suction, thus rejecting unwanted fluctuations in the tracing. This value was arbitrarily established for NS and applied to the entire sample, with no references to NNS. Thus, the present study conducted various tests to determine the suction threshold, until defining the calculation of each collection’s mean pressure signal added to the standard deviation. No fixed value was established for all NBs, considering that intersubject and intrasubject performance can vary greatly according to individual characteristics.

The analysis of the number of suctions showed that the second collection had higher values than the first and third ones. Also, the third collection recovered slightly in comparison with the first one.

A study^(17)^ reported decreases in the number of NNS in NBs, followed by stability in the suction rate, pointing to full recovery of initial performance after forced rest of approximately 1 minute.

A full-term NB’s suction pattern is characterized by more suctions per group and fewer and shorter pauses to rest^(2)^. Therefore, this is an important parameter to analyze, as its occurrence or interruption can characterize the stability and maturation of sucking behavior in NBs.

#### Suction groups

It was found that the second collection had a significantly shorter mean time to start suction groups than the first and third collections. Research indicates that difficulties in starting sucking may reflect possible changes in its mechanics^(2)^. Clinical observations also show that the delay in starting sucking behavior may be related to the baby's lack of stimulation, drowsiness during the assessment, and a test tip inadequately positioned in the oral cavity. Therefore, this is a relevant parameter to monitor the NB’s performance.

The analysis of the number and time of suction groups showed that the second collection had higher values than the first and third ones. Also, the values in the third collection recovered slightly in comparison with the first one.

A study^(15)^ with healthy full-term NBs measured each NB’s NNS for 12 minutes and then divided the recording into three periods of 4 minutes, observing that the duration of suction decreased from the first to the second period. The same study^(15)^ found that NBs under 24 hours old had longer sucking groups, with a quite variable pattern when compared to 2-to-3-day-old NBs.

Hence, the number and time of suction groups are relevant parameters, as they can vary according to the hours of life and the presence or absence of rest between measurements.

#### Suction frequency and period

The suction frequency data from the first collection are more distant from those from the third collection due to the gradual increase in values. As expected, the opposite occurs with the suction period, which also had a greater distance between the data from the first and third collections, caused, however, by the gradual decrease in values.

The progressive increase in frequency found in this study corroborates the finding^(15)^ that NNS frequency was significantly lower at the beginning of the analysis than in the subsequent periods.

A study^(18)^ reported that the NNS frequency is one suction per second, while another one^(4)^ reports 1.36 to 1.41 suctions per second. These references agree with the mean frequency and period of approximately one suction per second found in the present study.

Some authors^(19)^ have stated that suction frequency and amplitude and group duration have their patterns influenced by the NB’s gestational age, activity status, sex, and experience. Thus, suction frequency is an important parameter to be investigated and compared between different variables.

#### Suction amplitude and pressure

Pressure changes are responsible for triggering milk letdown in NS^(20)^. Therefore, quantitative data on the NB’s pressure during NNS are relevant to inform on performance and aptitude to start NS.

The analysis of suction pressure found mean minimum values of -8.43, -9.82, and -10.06 in the first, second, and third collections, respectively. Studies have found minimum peak values around -26.66 kPa^(21)^ and -24,52 kPa^(22)^. In the present research, the minimum peak found was -27.75 kPa. The mean maximum pressure values were -5.25, -6.36, and -6.24 in the first, second, and third collections, respectively. Studies^(21,23)^ have reported maximum pressures around -6.67 kPa. In both cases, the data found in the present study can be considered close to those reported in the literature.

The measures of central tendency also had extreme values (-1.39 kPa and -18.62 kPa) in the third collection, which influenced the significant increase in its standard deviation when compared to the other collections. In this case, the use of the prototype may have caused fatigue.

As for the means of all pressure peaks in the suction groups, the second collection had higher values than the first and third ones. Studies have reported mean pressures around -13.87 kPa^(21)^ and -15.2 kPa^(23)^.

It must be pointed out that the cited literature^(21-23)^ addresses NS measurement, which could justify possible differences in pressure values from the present study. NS values are described as greater than NNS values^(24)^. Moreover, different measurement methods may generate different data.

Still regarding pressure, the suction and pause behavior in some collections varied from 0 kPa. The characteristics indicated suction behavior at very high pressures while breathing pauses did not occur at around 0 kPa, thus tracing a sudden or gradual decline throughout the record. These data are relevant, and such parameters must be evaluated to compare the NB's performance in subjective and objective assessments. Authors stated that strong infant sucking has been associated with the mother’s nipple pain^(25)^. A study on the prevention of breastfeeding pain indicated that when the baby is hungrier when they start sucking, they are more likely to suck with excessive force^(26)^. Researchers also stated that poorly coordinated sucking can result from inadequate control of the oral structures and that sucking too strongly can injure the mother’s breasts^(2)^.

It is worth noting that NBs were assessed before breastfeeding. However, some NBs did not have pressure variation in the three collections. The highly variable pressure behavior during NNS is a pattern observed in clinical practice and may be related to several factors such as behavioral state, hunger, experience, and adaptation of oral motor control. Gestational age and sex are other factors that may influence results^(19)^ but were not analyzed in the present study. It is also believed that sucking behavior can be modified when assessed with a gloved finger or measuring prototype.

#### Pauses

The second collection had more pauses, which is expected because it had more suction groups. Hence, the pause time was also significantly shorter in the second collection since its sucking time was longer.

The sucking rhythm, characterized by suction sequences alternating with pauses, is essential for the NB’s sucking coordination and efficiency^(27)^. Therefore, the number and time of pauses must be analyzed in association with the number and time of suction groups in an instrument that aims to provide quantitative NNS information.

It is worth remembering that the present study assessed NBs with intact oral sensorimotor structures and functions and that some changes can modify the sucking pattern. For instance, research revealed that abnormal anatomical characteristics of the lingual frenulum influence tongue movement during NNS and suction rhythm during breastfeeding^(28)^.

Some authors have stated that the duration of pauses between sucking groups decreases with increasing maturation and sucking activity^(29)^. Therefore, the occurrence and duration of pauses are relevant parameters in quantitative NNS assessment.

#### Sporadic suctions

The second collection had significantly fewer sporadic suctions than the first and third collections.

Preterm NBs have a disorganized pattern with less sucking; as NNS subsequently develops, their experience increases the capacity to change the pattern^(18)^. Thus, although no difference was found in the present study, considering that only unaffected newborns were evaluated, the presence of sporadic sucking seems to be an important parameter to be investigated in high-risk babies.

### Reproducibility of parameters

The multiple comparisons between the three collections showed statistically significant differences between collections one and two and between two and three. Thus, it can be stated that collection three does not differ from collection one.

Thus, analyzing and comparing each variable separately, it was noticed that the second collection had more suction groups, more suctions, shorter time to start the suction groups, longer suction groups, fewer sporadic suctions, higher mean pressure with lower standard deviation, more pauses, and shorter pauses. These data indicate a greater readiness to initiate sucking behavior and significant maintenance of mean suction pressure and suction rhythm.

The comparisons suggest that the first measurement be used for training, while the second one is more reliable for analyzing the NB's performance. A study^(15)^ concluded that the sucking pattern changes during the analysis, indicating a sign of learning at the beginning, with gradually increasing frequency, and that the time of sucking without rest can decrease suction amplitudes in the final measurements.

### Computational parameter analysis

The agreement analysis with the ICC verified that the two independent evaluation methods (manual and computerized) had almost perfect agreement for all 12 parameters.

The high agreement, the guaranteed systematization of data extraction and analysis methods, the feasibility of applying the threshold to all records, and the significantly shorter analysis time (the manual took about 1 hour per graph, whereas the software took 1 minute per graph) demonstrate the effectiveness of the software programmed in Matlab^®^. Furthermore, other specific Matlab^®^ programming has already been described as a viable automatic system for objective suction assessment^(14)^.

Researchers also reported that when using independent evaluation methods, including visual identification and a suction detection analysis program, comparisons had close agreement between automated analysis and manual analysis^(15)^.

The literature highlighted the relevance of diagnostic methods for evaluating sucking parameters and suitability for oral feeding^(30)^. It also indicated the importance of measuring NNS to monitor measurements longitudinally and use them for teaching^(9)^.

The validation of an instrument to assess NNS in NBs positively influences the work of professionals who address breastfeeding, due to the standardization of assessments and the adequacy of therapeutic approaches.

The limitations of the research include the small sample and the lack of evaluation according to sex and gestational age since it was an initial exploratory study. Future studies should analyze these variables, improve the device design, assess preterm NBs, assess NBs with lingual frenulum changes, analyze fatigue behavior throughout measurements, use video recording during evaluations, and develop a protocol for visual classification of the tracing so that subjective and objective evaluation can later be compared.

## CONCLUSION

The number of suction groups, number of suctions, time to start the suction groups, suction group time, suction frequency, number of sporadic suctions, minimum pressure, mean pressure, maximum pressure, number of pauses, and time of pauses proved to be relevant to assess NNS.

The data suggests a training behavior in the first collection and a greater readiness to start suction and maintain mean suction pressure and a significant suction rhythm in the second one.

Thus, the use of the Matlab^®^ program to extract and analyze NNS parameters proved to be feasible and effective, and the analysis using the program showed high agreement with the manual evaluation.

## References

[1] Caetano LC, Fuginaga CI, Scochi CG (2003). Non-nutritive sucking in pre-term infants: a bibliographic study. Rev Lat Am Enfermagem.

[2] Glass RP, Wolf LS (1994). A global perspective on feeding assessment in the neonatal intensive care unit. Am J Occup Ther.

[3] Bernbaum JC, Pereira GR, Watkins JB, Peckham GJ (1983). Nonnutritive sucking during gavage feeding enhances growth and maturation in premature infants. Pediatrics.

[4] Neiva FCB, Leone C, Leone CR (2008). Non-nutritive sucking scoring system for preterm newborns. Acta Paediatr.

[5] Palmer MM, Crawley K, Blanco IA (1993). Neonatal oral-motor assessment scale: a reability study. J Perinatol.

[6] Fuginaga CI (2007). Reliability of an instrument to assess the readiness of preterm infants for oral feeding. Pró-Fono R Atual Cient.

[7] Xavier C (1995). Validação do conteúdo de um instrumento para avaliação da prontidão do prematuro para início da alimentação oral. Pró-Fono R Atual Cient.

[8] Prieto CR, Cardenas H, Salvatierra AM, Boza C, Montes CG, Croxatto HB (1996). Sucking pressure and its relationship to milk transfer during breastfeeding in humans. J Reprod Fertil.

[9] Lau C, Kusnierczyk I (2001). Quantitative evaluation of infant’s nonnutritive and nutritive sucking. Dysphagia.

[10] McGowan JS, March RR, Fowler SM, Levy SE, Stallings VA (1991). Developmental patterns of normal nutritive sucking in infants. Dev Med Child Neurol.

[11] Medoff-Cooper B, Bilker W, Kaplan JM (2010). Sucking patterns and behavioral state in 1- and 2-day-old full-term infants. J Obstet Gynecol Neonatal Nurs.

[12] Tamilia E, Delafield J, Fiore S, Taffoni F (2014). An automatized system for the assessment of nutritive sucking behavior in infants: a preliminary analysis on term neonates. Annu Int Conf IEEE Eng Med Biol Soc..

[13] White-Traut R, Rankin K, Lucas R, Shapiro N, Medoff-Cooper B (2013). Evaluating sucking maturation under two pressure thresholds. Early Hum Dev.

[14] Tamilia E, Formica D, Scaini A, Taffoni F (2016). An automated system for the analysis of newborns oral-motor behavior. IEEE Trans Neural Syst Rehabil Eng.

[15] Hafström M, Lundquist C, Lindecrantz K, Larsson K, Kjellmer I (1997). Recording non-nutritive sucking in the neonate: description of an automatized system for analysis. Acta Paediatr.

[16] Ramos CAV (2015). Protótipo de instrumento para avaliação de sucção não-nutritiva em recém-nascidos.

[17] Levin G, Kaye H (1966). Work decrement and rest recovery during non-nutritive sucking in the human neonate. J Exp Child Psychol.

[18] Silva RNM, Lopes SMB, Lopes JMA (1999). Follow up do recém-nascido de alto risco..

[19] Lundqvist C, Hafström M (1999). Non-nutritive sucking in full-term and preterm infants studied at term conceptional age. Acta Paediatr.

[20] Hernandez AM, Hernandez AM, Marchesan I (2001). Fonoaudiologia em berçário normal e de risco..

[21] Sameroff AJ (1968). The components of sucking in the human newborn. J Exp Child Psychol.

[22] Colley JR, Creamer B (1958). Sucking e swallowing in infants. BMJ.

[23] Geddes DT, Kent J, Mitoulas L, Hartmann P (2008). Tongue movement and intra-oral vacuum in breastfeeding infants. Early Hum Dev.

[24] Wolf PH (1968). The serial organization of sucking in the young infant. Pediatrics.

[25] McClellan HL, Geddes DT, Kent JC, Garbin CP, Mitoulas LR, Hartmann PE (2008). Infants of mothers with persistent nipple pain exert strong sucking vacuums. Acta Paediatr.

[26] Biancuzzo M (2000). Sore nipples: prevention and problem solving..

[27] Vice FL, Bosma JF, Gewolb IH (2001). Developmental changes in respiratory patterning and synchronization during rhythmic suckle feeding in premature infants. Pediatr Res Baltimore..

[28] Martinelli RLC, Marchesan IQ, Berretin-Felix G (2013). Lingual frenulum evaluation protocol for infants: relationship between anatomic and functional aspects. Rev CEFAC.

[29] Hafström M, Kjellmer K (2000). Non-nutritive sucking in the healthy pre-term infant. Early Hum Dev.

[30] Costa SP, van den Engel-Hoek L, Bos AF (2008). Sucking and swallowing in infants and diagnostic tools. J Perinatol.

