# Supplementary material for: Characterization of parameters for the analysis of objective measures of non-nutritive sucking of newborns
Source: Codas. 2024 May 31;36(4):e20230149. doi: 10.1590/2317-1782/20242023149en (PMC11189154; doi:10.1590/2317-1782/20242023149en)
Supplement: Translation

# Translation: Caracterização de parâmetros para análise de medidas objetivas da sucção não-nutritiva de recém-nascidos

**DOI:** 10.1590/2317-1782/20242023149pt

**Authors:** Bárbara Generoso Santos de Matos Sales, Renata Maria Moreira Moraes Furlan, Camila Alexandra Vilaça Ramos, Narciso Sena Fracaroli, Estevam Barbosa de Las Casas, Andréa Rodrigues Motta

**Affiliations:** 1 Programa de Pós-graduação em Ciências Fonoaudiológicas, Universidade Federal de Minas Gerais – UFMG - Belo Horizonte (MG), Brasil.; 2 Departamento de Fonoaudiologia, Faculdade de Medicina, Universidade Federal de Minas Gerais – UFMG - Belo Horizonte (MG), Brasil.; 3 Programa de Pós-graduação em Engenharia Mecânica, Universidade Federal de Minas Gerais – UFMG - Belo Horizonte (MG), Brasil.; 4 Universidade Federal de Minas Gerais – UFMG - Belo Horizonte (MG), Brasil.; 5 Departamento de Engenharia de Estruturas, Escola de Engenharia, Universidade Federal de Minas Gerais – UFMG - Belo Horizonte (MG), Brasil.

**Keywords:** Sucção Não-nutritiva, Comportamento de Sucção, Recém-nascido, Equipamentos de Medição, Amamentação

## Abstract

**Objetivo:**

Propor uma metodologia de análise dos dados gerados por um instrumento de medição da pressão de sucção não-nutritiva do recém-nascido.

**Método:**

Estudo observacional analítico, transversal com dados de 24 recém-nascidos a termo sem comprometimentos. Após a avaliação clínica foram analisadas três coletas de cada neonato empregando-se o referido instrumento, com duração de 2 minutos e intervalo de 2 minutos entre elas. Os parâmetros definidos foram extraídos por meio de um programa desenvolvido em Matlab®. Os resultados foram obtidos pela análise e comparação de 12 variáveis ao nível de confiança de 5%. Foi realizada ainda comparação das análises manual e computadorizada por meio do coeficiente de correlação intraclasse.

**Resultados:**

A comparação múltipla entre os três momentos de coleta, mostrou que as diferenças estatísticas significantes ocorreram entre as coletas um e dois e dois e três. Ao se analisar e comparar cada variável separadamente, notou-se que a segunda coleta apresentou: maior número de grupos de sucção, maior número de sucções, menor tempo para iniciar os grupos de sucção, maior tempo de grupos de sucção, menor número de sucções esporádicas, valores de pressão média maiores e com menor desvio padrão, maior número de pausas com tempo de pausas menor. O coeficiente de correlação intraclasse revelou concordância quase perfeita para os 12 parâmetros avaliados.

**Conclusão:**

As 12 variáveis analisadas mostram-se relevantes, especialmente na segunda coleta. O programa em Matlab® mostrou-se viável e eficaz na extração e análise dos parâmetros, apresentando alta concordância quando comparado à avaliação manual.

## INTRODUÇÃO

Espera-se que um neonato a termo sem alterações já possua ao nascer condições de se alimentar por via oral sem prejuízos de suas funções vitais. Para tanto, as funções de sucção, deglutição e respiração devem ocorrer de forma coordenada e harmônica, e características como vedamento labial, movimentos adequados de língua e mandíbula, ritmo de sucção e pausas devem ser observadas^(1,2)^.

Existem dois tipos de sucção, a não-nutritiva (SNN) em que são realizados movimentos de sucção sem a introdução de líquido na cavidade oral e a sucção nutritiva (SN) em que o bebê realiza a retirada do líquido do seio ou recipiente para a cavidade oral, com o objetivo de se alimentar^(1)^. Os dois tipos de sucção compartilham parâmetros que podem ser descritos em termos de padrão de sucção, taxa de sucção, ritmo e quantidade de pressão, entretanto são distintos os surtos de sucção e as pausas^(2)^.

A sucção não-nutritiva integra um conjunto de habilidades necessárias ao bebê para sua maturação e desenvolvimento^(1)^ e traz muitos benefícios ao recém-nascido (RN), como a redução da dor quando submetido a procedimentos clínicos dolorosos e a diminuição do choro. A estimulação da SNN auxilia na adequação da musculatura oral e estimula a função motora gástrica facilitando o ganho de peso, o que diminui o tempo de internação hospitalar^(3)^.

Além disso, a SNN é preditiva do início da alimentação oral, indicando a possibilidade da realização da sucção nutritiva^(4)^. Fatores como prematuridade, alterações neurológicas e má formações craniofaciais podem influenciar o comportamento de sucção de recém-nascidos, acarretando em dificuldades com a alimentação segura e prejuízos no desenvolvimento das funções estomatognáticas do bebê^(1)^.

O fonoaudiólogo é o profissional capacitado para realizar a avaliação e o treinamento da SNN no Brasil, entretanto existem atualmente poucos protocolos e escalas para o uso clínico. Escalas qualitativas como NEIVA^(4)^, Neonatal Oral-Motor Assessment Scale (NOMAS)^(5)^, FUGINAGA^(6)^ e XAVIER^(7)^ são validadas e baseiam-se na observação descritiva do comportamento motor oral dos bebês, avaliando também outros aspectos do desenvolvimento global do neonato. Essas escalas têm em comum a classificação categórica de seus achados, como comportamentos presentes, ausentes, fracos, fortes, adequados, inadequados, dentre outros, e suas respostas podem variar de acordo com a experiência do avaliador.

Existem poucos estudos sobre a utilização de instrumentos para a avaliação quantitativa da sucção^(8-15)^, e a maioria deles tem o enfoque na sucção nutritiva, explorando pouco os achados da sucção não-nutritiva.

Alguns destes estudos quantitativos não revelam a forma de análise dos dados detectados e registrados pelo equipamento de medição. Dentre os métodos relatados estão o uso do julgamento da mãe ao comportamento de sucção associada à quantificação dos padrões^(8)^; a comparação de avaliações cegas entre observadores^(9)^; a menção de programas ou *softwares* personalizados para extração de dados^(10-12)^; o uso do *software* Matlab ®^(13,14)^ para processamento dos sinais biológicos e a aplicação de métodos avaliativos independentes para comparação^(15)^.

Desta forma, a existência de um número reduzido de estudos quantitativos, algumas vezes somada à abordagem rápida e superficial da forma de tratamento dos dados, contribuem para a dificuldade de sistematização de métodos de análise do comportamento de SNN.

Diante desta necessidade, um sistema de medição^(16)^ foi proposto pelo Grupo de Engenharia Biomecânica da Universidade Federal de Minas Gerais, com o objetivo de auxiliar na avaliação clínica da sucção não-nutritiva. Entretanto, não apenas o desenvolvimento deste instrumento, mas a definição de parâmetros para análise dos dados gerados por ele e a apresentação de uma metodologia de uso que possa ser sistematizada torna-se fundamental para a normatização e validação do equipamento.

O uso de um método quantitativo de avaliação da SNN representará um avanço científico, contribuindo na prática clínica fonoaudiológica, visto que os profissionais envolvidos nos casos poderão valer-se da mesma óptica de avaliação, influenciando positivamente a discussão de condutas terapêuticas.

Desta forma, o presente estudo teve como objetivo propor uma metodologia de análise dos dados gerados por um instrumento de medição da pressão de sucção não-nutritiva do recém-nascido.

## MÉTODO

Trata-se de um estudo do tipo observacional analítico, com delineamento transversal, realizado a partir dos dados coletados para a pesquisa de Ramos^(16)^, após aprovação do Comitê de Ética em Pesquisa da instituição de origem, sob o número 32078014.0.0000.5149.

A referida pesquisa^(16)^ desenvolveu um método de avaliação instrumental da sucção não-nutritiva (Figura 1) constituído por uma ponta probatória (componente introduzido na cavidade oral), conectores (que ligam a ponta probatória ao sensor de vácuo), um sensor de vácuo - Sensor CRM-5-10 Sensum (que capta a pressão negativa e gera um sinal tratado, transmitido, processado e armazenado em meio digital) e um sistema de armazenamento e análise de dados (NI USB 6008, National Instruments, Austin, TX).

**Figura 1:** Instrumento de medição da sucção não-nutritiva^(16)^

Fonte: Ramos^(16)^

Após a calibração do sensor, realizada no Laboratório Isaac Newton da Gerência de Metrologia do CITSF – Campus CETEC, obteve-se uma incerteza expandida de medição relativa máxima de 17%, no ponto de -5 kPa.

As medições foram realizadas no Hospital Sofia Feldman - Fundação de Assistência Integral à Saúde. Participaram da pesquisa primária^(16)^ 30 bebês a termo com idade gestacional igual ou superior a 37 semanas, peso adequado ao nascimento, com estruturas e funções do sistema sensório motor oral íntegras, sem alterações do frênulo lingual, ausência de más formações craniofaciais e de alterações neurológicas, clínicas e respiratórias; com capacidade de alimentação preservada e em aleitamento materno exclusivo, independentemente do sexo, horas de vida ou tipo de parto. Os responsáveis pelos RNs assinaram o Termo de consentimento Livre e Esclarecido.

Os bebês foram submetidos a dois tipos de avaliação, pelo mesmo avaliador, com mais de 10 anos de experiência na área. Inicialmente foi aplicado o protocolo qualitativo da sucção não-nutritiva^(4)^ e em sequência à avaliação objetiva da SNN com o instrumento elaborado (Figura 1).

Durante os dois métodos de avaliação realizados na pesquisa primária^(16)^ os RNs foram avaliados no colo da pesquisadora, posicionados em supino, apoiados na região cervical com a cabeça e o pescoço mais elevados em relação ao restante do corpo. Para a avaliação qualitativa o dedo mínimo enluvado da pesquisadora foi introduzido entre os lábios do RN e mantido em sua cavidade oral por um período de dois minutos. Posteriormente para a avaliação objetiva, o posicionamento do bebê foi mantido como descrito acima e o dedo enluvado foi substituído pela ponta probatória do instrumento (Figura 1) que foi introduzida entre os lábios do RN viabilizando a captação da pressão de sucção.

Após a avaliação clínica, a pesquisadora realizou três medições para cada RN, com tempo de dois minutos de duração e intervalo de dois minutos entre cada medição para descanso.

Os critérios de inclusão da pesquisa primária^(16)^, além dos acima descritos, foram o RN ter apresentado pressão de sucção adequada durante a avaliação clínica da sucção não-nutritiva realizada por meio do protocolo de avaliação subjetiva^(4)^ e assinatura do termo de consentimento livre esclarecido pelo responsável. Os critérios de exclusão^(16)^ foram RNs que, durante as avaliações mantiveram estados comportamentais de choro e irritação ou apresentaram ausência de sucção durante a medição.

A fim de possibilitar a análise dos registros obtidos na pesquisa de Ramos, 2015^(16)^, fez-se necessário a definição de novos critérios de exclusão para o presente estudo.

Foram considerados como critérios de exclusão RNs que apresentassem um ou mais registros com interferências (ruídos repetidos e contínuos ao longo do tempo), que não apresentassem no mínimo um grupo de sucção por coleta, que não atingissem o tempo de dois minutos por coleta, bem como apresentassem a duplicação de traçados (dados de pressão e tempo replicados no momento em que os arquivos foram gerados no sistema de armazenamento do equipamento^(16)^).

Dessa forma, utilizando os critérios de exclusão da pesquisa primária e do presente estudo, a amostra final da pesquisa foi constituída por 24 RNs, 15 (62,5%) do sexo masculino e nove (37,5%) do feminino, com média de idade em horas de 19,5; média de idade gestacional de 39,1 semanas; média do peso ao nascimento de 3.307,08 gramas e média de pontuação da avaliação subjetiva^(4)^ de 75,50.

Para possibilitar o início da análise, foi necessário estabelecer um valor de pressão que indicasse quais sinais poderiam ser contabilizados como sucções. Esse limiar impede, principalmente, que interferências e possíveis movimentações sejam indevidamente assumidas como sucções.

Assim, testes foram realizados para determinar um valor que não apenas indicasse um limiar de pressão para contabilização das sucções, mas que também fosse capaz de considerar o desempenho individual de cada RN. Os testes calculavam a média de pressão de todo o traçado somada a variações do seu desvio padrão.

O cálculo dos intervalos de confiança evidenciou que a média do sinal de pressão somada ao desvio padrão, poderia ser assumida como o limiar. Desconsiderando assim as pequenas interferências e possibilitando a variação de acordo com o desempenho exercido pelo RN a cada medição.

Desta forma, determinou-se como sucções os sinais que ultrapassavam o limiar definido como a média de todo sinal de pressão somada ao valor do desvio padrão. Para diferenciação dos eventos de sucção, duas sucções muito próximas foram contabilizadas como eventos independentes caso as duas variações de pressão excedessem o limiar estabelecido anteriormente. Caso contrário as duas deflexões foram contabilizadas como partes do mesmo evento, com o valor de pico correspondendo à maior deflexão^(12)^.

Considerando-se os parâmetros investigados pela literatura^(2,4,9-12,14,15)^ e com o objetivo de sistematizar a extração das variáveis e reduzir a possibilidade de aferição de valores inconsistentes; após a determinação do limiar de pressão, os demais parâmetros foram definidos:

1. Número de grupos de sucção: Os grupos de sucção são caracterizados pela presença de três ou mais sucções com intervalos de tempo menor que três segundos entre as sucções^(4)^ (Figura 2).

**Figura 2:** Parâmetros definidos para o estudo

Legenda: A = pausa, B = grupo de sucção, C = sucção esporádica, D = limiar de sucção
2. Número de sucções: Número de sucções do traçado que integram grupos de sucção.
3. Tempo para iniciar os grupos de sucção: Tempo gasto pelo recém-nascido até o início do primeiro grupo de sucção (s).
4. Tempo dos grupos de sucção: Tempo de realização do grupo de sucção (s).
5. Frequência de sucção (f): número de sucções realizadas por segundo, é o inverso do período (n/s).
6. Período de sucção: intervalo entre dois picos de sucção consecutivos no grupo (1/f).
7. Número de sucções esporádicas: eventos de sucção isolada que não integram nenhum grupo de sucção (Figura 2).
8. Valor de pressão mínima: maior valor de amplitude de sucção dos grupos (kPa).
9. Valor de pressão média: média dos picos de pressão dos grupos (kPa).
10. Valor de pressão máxima: menor valor de amplitude de sucção dos grupos (kPa).
11. Número de pausas: As pausas são intervalos de tempo maior ou iguais a três segundos sem a presença de um grupo de sucção^(4)^ (Figura 2).
12. Tempo de pausas: Tempo de realização das pausas (s).

Para a análise dos registros, o Grupo de Engenharia Biomecânica da UFMG desenvolveu um programa em Matlab®. Optou-se por um tratamento inicial com um filtro de Resposta Finita ao Impulso (FIR) do tipo passa-baixa. Criou-se uma curva auxiliar para simplificar o processo de identificação dos picos, obtida por meio da correção da curva original (Figura 3). Então, aplicou-se um tratamento baseado em um método proposto^(12)^, com as devidas adaptações, para a filtragem de pontos não relevantes identificados inicialmente. Desta forma, com todos os máximos conhecidos na curva original (Figura 4), fez-se o reconhecimento dos grupos e realizou-se operações para a determinação das variáveis de interesse.

**Figura 3:** Gráfico com correção

**Figura 4:** Gráfico sem correção

Após a programação do *software* as variáveis extraídas foram transcritas e organizadas em um banco de dados do programa Excel.

Para a verificação de concordância entre as análises manual e computadorizada, foram sorteados seis RNs (25% da amostra total) aleatoriamente entre aqueles que possibilitavam a análise manual para posterior análise de concordância.

As variáveis de interesse foram caracterizadas por meio de medidas de tendência central e dispersão. Os dados foram analisados no *software* SPSS, versão 21.0 por meio dos testes não paramétricos de Friedman e Wilcoxon a 5% de significância para comparação dos valores de cada variável entre as três coletas. Para verificar a concordância entre as análises realizadas por dois métodos distintos de avaliação, foi utilizado o coeficiente de correlação intraclasse (CCI).

## RESULTADOS

A análise descritiva de tópicos do Formulário de Avaliação da Sucção não-nutritiva^(4)^ revelou que a maioria dos recém-nascidos nasceram de parto do tipo normal (75,0%); a maioria sempre iniciou facilmente a sucção (70,8%); sempre apresentou força (70,8%) e ritmo (66,7%) de sucção e a maioria nunca apresentou sinais de estresse (83,3%) durante a avaliação.

Na Tabela 1 são apresentadas as medidas de tendência central e dispersão das variáveis relacionadas aos eventos de sucção: número de grupos de sucção, número de sucções, tempo para iniciar grupos de sucção, tempo dos grupos de sucção, frequência de sucção, período de sucção e número de sucções esporádicas.

**Tabela 1:** Medidas de tendência central e dispersão das variáveis de interesse relacionadas aos eventos de sucção

	N° de grupos de sucção	N° de sucções	Tempo para iniciar grupos de sucção (s)	Tempo dos grupos de sucção (s)	Frequência de sucção (n/s)	Período de sucção (s)	Nº de sucções esporádicas
**Coleta 1**							
Média	4,21	29,63	28,60	36,48	0,84	1,29	4,46
DP	2,04	19,37	25,77	22,44	0,17	0,32	2,73
Mínimo	1,00	5,00	1,30	4,40	0,53	0,88	0,00
Mediana	4,00	27,50	17,00	34,20	0,90	1,15	5,00
Máximo	8,00	66,00	93,00	74,50	1,14	1,97	10,00
**Coleta 2**							
Média	5,21	40,45	16,33	48,73	0,85	1,24	3,04
DP	1,74	18,91	12,39	20,94	0,10	0,19	2,42
Mínimo	2,00	11,00	0,90	13,50	0,59	1,01	0,00
Mediana	6,00	40,00	13,05	52,10	0,84	1,22	2,00
Máximo	8,00	73,00	44,00	84,70	1,02	1,88	9,00
**Coleta 3**							
Média	4,50	33,25	22,68	39,79	0,90	1,18	3,54
DP	1,74	19,63	22,07	21,06	0,14	0,21	3,50
Mínimo	1,00	8,00	0,70	11,10	0,57	0,89	0,00
Mediana	5,00	33,50	14,30	36,60	0,89	1,14	2,50
Máximo	7,00	77,00	92,10	84,20	1,14	1,76	13,00

Legenda: N° = número; DP = desvio padrão; s = segundos; n/s = número de sucções por segundo

Na Tabela 2 são apresentadas as medidas de tendência central e dispersão das variáveis relacionadas à pressão e à pausa: pressão mínima, pressão média, pressão máxima, números de pausas e tempo de pausas.

**Tabela 2:** Medidas de tendência central e dispersão das variáveis de interesse relacionadas à pressão em kPa e à pausa

	Pressão mínima	Pressão Média	Pressão máxima	N° de pausas	Tempo de pausas (s)
**Coleta 1**					
Média	- 8,43	- 7,04	- 5,25	4,50	83,52
DP	4,08	3,71	3,29	1,77	22,44
Mínimo	- 17,46	- 15,07	- 13,48	2,00	45,50
Mediana	- 9,21	- 6,69	- 4,56	5,00	85,80
Máximo	- 2,95	- 2,18	- 1,73	8,00	115,60
**Coleta 2**					
Média	- 9,82	- 8,49	- 6,36	5,46	71,28
DP	4,15	3,65	3,39	1,53	20,94
Mínimo	- 15,93	- 13,82	- 11,97	2,00	35,30
Mediana	- 11,20	- 9,43	- 6,24	5,00	67,90
Máximo	- 3,31	- 2,89	- 1,92	8,00	106,50
**Coleta 3**					
Média	- 10,06	- 8,47	- 6,24	4,83	80,21
DP	4,79	4,10	3,77	1,52	21,06
Mínimo	- 18,62	- 15,25	- 13,78	2,00	35,80
Mediana	- 10,69	- 9,16	- 6,77	5,00	83,40
Máximo	- 2,42	- 2,19	- 1,39	8,00	108,90

Legenda: N° = número; DP = desvio padrão; s = segundos

Na Tabela 3 é apresentada a comparação de cada variável entre os três momentos de coleta, por meio do teste de Friedman, revelando associações com significância estatística. A tabela também apresenta o teste de Wilcoxon que identifica em quais pares as diferenças ocorreram.

**Tabela 3:** Comparação dos momentos dois a dois

**Variáveis**	**Valor-p****1**	**Comparações múltiplas** **2** **entre as coletas**		
		**1 x 2**	**2 x 3**	**3 x 1**
N° de grupos de sucção	0,057	-	-	-
N° de sucções	0,005*	0,007*	0,052	1,000
Tempo para iniciar grupos de sucção	0,115	-	-	-
Tempo dos grupos de sucção	0,005*	0,018*	0,012*	1,000
Frequência de sucção	0,034*	0,582	0,028*	0,582
Período de sucção	0,093	-	-	-
Nº de sucções esporádicas	0,213	-	-	-
Pressão mínima	0,100	-	-	-
Pressão média	0,001*	0,001*	0,337	0,130
Pressão máxima	0,331	-	-	-
N° de pausas	0,017*	0,028*	0,250	1,000
Tempo de pausas	0,005*	0,018*	0,012*	1,000

1 Teste de Friedman;

2 Teste de Wilcoxon;

* p≤0,05

Legenda: N° = número

Na Tabela 4, por meio do coeficiente de correlação intraclasse (CCI), é apresentada a análise de concordância entre as 12 variáveis de interesse, extraídas pelos dois métodos de avaliação (manual e computadorizado). A análise indicou concordância quase perfeita para todas as variáveis.

**Tabela 4:** Análise de concordância entre avaliações das 12 variáveis de interesse

**Variáveis**		**Análise manual**											
		**1^a^**	**2^a^**	**3^a^**	**4^a^**	**5^a^**	**6^a^**	**7^a^**	**8^a^**	**9^a^**	**10^a^**	**11^a^**	**12^a^**
Análise software	1^a^	0,986	-	-	-	-	-	-	-	-	-	-	-
	2^a^	-	0,997	-	-	-	-	-	-	-	-	-	-
	3^a^	-	-	0,995	-	-	-	-	-	-	-	-	-
	4^a^	-	-	-	0,967	-	-	-	-	-	-	-	-
	5^a^	-	-	-	-	0,934	-	-	-	-	-	-	-
	6^a^	-	-	-	-	-	0,954	-	-	-	-	-	-
	7^a^	-	-	-	-	-	-	0,900	-	-	-	-	-
	8^a^	-	-	-	-	-	-	-	0,999	-	-	-	-
	9^a^	-	-	-	-	-	-	-	-	0,997	-	-	-
	10^a^	-	-	-	-	-	-	-	-	-	0,995	-	-
	11^a^	-	-	-	-	-	-	-	-	-	-	0,964	-
	12^a^	-	-	-	-	-	-	-	-	-	-	-	0,968

Coeficiente de correlação intraclasse

Legenda: 1ª Número de grupos de sucção; 2ª Número de sucções; 3ª Tempo para iniciar grupos de sucção; 4ª Tempo dos grupos de sucção; 5ª Frequência de sucção; 6ª Período de sucção; 7ª Número de sucções esporádicas; 8ª Pressão mínima; 9ª Pressão média; 10ª Pressão máxima; 11ª Número de pausas; 12ª Tempo de pausas

## DISCUSSÃO

O presente trabalho teve como base os dados da pesquisa primária de desenvolvimento de um instrumento para avaliação de sucção não-nutritiva em recém-nascidos^(16)^ realizada pelo Grupo de Engenharia Biomecânica da UFMG. Para análise pretendida foram comparadas as variáveis encontradas na avaliação prévia subjetiva e nas três coletas da avaliação quantitativa de SNN de 24 recém-nascidos (15 meninos e nove meninas) a fim de propor uma forma de análise dos registros.

### Caracterização dos parâmetros

Como apresentado na literatura o comportamento de sucção pode ser descrito em padrões e taxas de sucção, ritmo de sucção e pausa e quantidade de pressão alcançada pelo RN^(2)^.

Eventos como número, duração e frequência de sucções, grupos de sucções, pausas, pressão exercida e coordenação entre sucção, deglutição e respiração são monitoradas simultaneamente em uma avaliação clínica de rotina^(9)^. Portanto, a possibilidade de mensuração objetiva e revisão posterior destes dados torna-se essencial^(9)^.

Assim, a definição dos parâmetros do presente estudo, foi baseada nos comportamentos investigados em escalas qualitativas validadas e em pesquisas quantitativas da SN e SNN. A importância da mensuração objetiva destas medidas, a possibilidade de análise posterior dos registros e a padronização de um método avaliativo, justificam a escolha destes parâmetros e a sua inclusão na análise computacional dos dados realizados por esta pesquisa.

#### Sucções

Um estudo objetivo sobre os padrões de desenvolvimento da sucção nutritiva^(10)^ estabeleceu um limiar de -16 mmHg como critério para detecção de sucções, rejeitando assim flutuações indesejadas do traçado. Considerando que esse valor foi estabelecido arbitrariamente para a SN e aplicado para toda a amostra, bem como a ausência de referências para a SNN, no presente trabalho, para a determinação do limiar de sucção, vários testes foram realizados até a definição do cálculo de média do sinal de pressão somada ao valor do desvio padrão de cada coleta analisada. Optou-se pelo não estabelecimento de um valor fixo para todos os RNs, por considerar que o desempenho inter e intra sujeito pode variar bastante de acordo com as características individuais.

Ao se analisar a variável número de sucções foi possível observar que a segunda coleta apresentou valores maiores quando comparada à primeira e terceira coletas. É importante notar que a terceira coleta apresentou uma leve retomada de valores quando comparada à primeira.

Um estudo^(17)^ relatou decréscimos no número de SNN dos neonatos, seguida de estabilidade da taxa de número de sucções, apontando a ocorrência de recuperação total do desempenho inicial após o repouso forçado de aproximadamente um minuto.

O padrão de sucção de um RN a termo é caracterizado por um maior número de sucções por grupos, com menos e mais breves períodos de pausa para descanso^(2)^. Desta forma, este é um importante parâmetro a ser analisado, pois sua ocorrência ou interrupção pode caracterizar a estabilidade e maturação do comportamento de sucção em neonatos.

#### Grupos de sucção

Em relação à variável tempo para iniciar os grupos de sucção, foi possível observar que a segunda coleta apresentou uma média de tempo significativamente menor quando comparada à primeira e terceira coleta. Uma pesquisa indica que dificuldades no início da sucção podem refletir possíveis alterações em sua mecânica^(2)^. As observações clínicas mostram ainda que a demora para iniciar a o comportamento de sucção pode estar relacionada à pouca estimulação do bebê, estado de sonolência durante a avaliação e posicionamento inadequado da ponta probatória na cavidade oral. Desta forma, este é um parâmetro relevante para acompanhar o desempenho do RN.

Ao se analisar as variáveis número de grupos de sucção e tempo dos grupos de sucção foi possível observar que a segunda coleta apresenta valores maiores quando comparada à primeira e terceira coletas. É importante notar que a terceira coleta apresentou uma leve retomada de valores quando comparada à primeira.

Um estudo^(15)^ com RNs a termo saudáveis realizou a medição de SNN durante 12 minutos para cada RN e posteriormente dividiu o registro em três períodos de quatro minutos, observando que a duração dos tempos de sucção diminuiu entre o primeiro e o segundo período. O mesmo estudo^(15)^ verificou que neonatos com menos de 24 horas de vida apresentaram maior duração dos grupos de sucção, com um padrão bastante variável quando comparados a RNs de dois e três dias de vida.

É possível notar que o número de grupos de sucção e o tempo de ocorrência destes grupos são parâmetros relevantes, pois podem variar de acordo com as horas de vida e a presença ou ausência de descanso entre as medições.

#### Frequência e período de sucção

Em relação à variável frequência de sucção foi possível observar que os dados da primeira coleta são mais distantes dos dados da terceira coleta devido ao aumento gradativo dos valores encontrados. Como esperado, o inverso ocorre com a variável período de sucção, que também apresentou uma distância maior entre os dados da primeira e a terceira coleta, entretanto, ocasionada pela diminuição gradativa dos valores encontrados.

O aumento progressivo da frequência encontrado neste estudo corrobora o achado^(15)^ em que a frequência de SNN apresentou um valor significativamente menor no início da análise em comparação com os períodos seguintes.

Um estudo^(18)^ informou que o valor de frequência da SNN é de uma sucção por segundo e outro trabalho^(4)^ relata valores de 1,36 a 1,41 sucções por segundo. Estas referências concordam com os valores médios de frequência e período de aproximadamente uma sucção por segundo encontrados no presente estudo.

Autores^(19)^ afirmaram que a frequência de sucção, assim como a amplitude das sucções e a duração dos grupos, tem seus padrões influenciados pela idade gestacional, estado de atividade, sexo e a experiência do RN. Desta forma, a frequência de sucção representa um importante parâmetro a ser investigado e comparado entre diferentes variáveis.

#### Amplitude e pressão de sucção

As mudanças de pressão são responsáveis por desencadear a retirada do leite na SN^(20)^, desta forma, dados quantitativos sobre a pressão realizada pelos neonatos durante a SNN são relevantes para informar sobre o desempenho e a aptidão para iniciar a SN.

Ao se analisar as variáveis de pressão de sucção, os valores médios das pressões mínimas foram de -8,43; -9,82 e -10,06 na primeira, segunda e terceira coletas, respectivamente. Pesquisas encontraram valores de picos mínimos em torno de -26,66 kPa^(21)^ e -24,52 kPa^(22)^. Na presente pesquisa o pico mínimo encontrado foi de -27,75 kPa. Os valores médios das pressões máximas foram de - 5,25; - 6,36 e - 6,24 na primeira, segunda e terceira coleta respectivamente. Estudos^(21,23)^ relataram a ocorrência de pressão máxima por volta de -6,67 kPa. Nos dois casos é possível observar que os dados encontrados podem ser considerados próximos aos relatados na literatura.

As medidas de tendência central evidenciaram ainda a ocorrência de valores extremos (-1,39 kPa e -18,62 kPa) na terceira coleta, o que influenciou no aumento significativo do seu desvio padrão quando comparada às outras coletas. Pode-se considerar nesse caso que pode ter ocorrido cansaço com o uso do protótipo.

Quanto às médias de todos os picos de pressão dos grupos de sucção, a segunda coleta apresentou valores mais elevados em relação à primeira e à terceira. Estudos relataram médias de pressão ocorrendo por volta de -13,87 kPa^(21)^ e -15,2 kPa^(23)^.

É importante ressaltar que a literatura citada^(21-23)^ trata da medição da sucção nutritiva, o que poderia justificar possíveis diferenças de valores de pressão quando comparados ao presente estudo. Os valores de SN são descritos como maiores que os da SNN^(24)^. Além disso, métodos de medição diferentes podem gerar dados diferentes.

Ainda com relação à pressão, algumas coletas analisadas mostraram variação do comportamento de sucção e pausas em relação à pressão de 0kPa. As características apontavam um comportamento de sucção com pressões bastante elevadas, em que a pausa respiratória não era realizada em torno de 0 kPa. Desta forma, traçando um declínio brusco ou gradativo ao longo do registro. Esses dados são relevantes e tais parâmetros devem ser avaliados para a comparação de desempenho do RN nas avaliações subjetiva e objetiva. Autores afirmaram que a forte sucção infantil tem sido associada à dor nos mamilos da lactante^(25)^. Um estudo sobre a prevenção de dor durante a amamentação indicou que ao iniciar a sucção com mais fome, o bebê tem mais chances de sugar com força excessiva^(26)^. Pesquisadores afirmaram também que a sucção mal coordenada pode resultar do controle inadequado das estruturas orais, bem como a realização de sucção muito forte pode causar ferimento nas mamas da lactante^(2)^.

Cabe ressaltar que as avaliações foram realizadas antes das mamadas, entretanto, alguns RNs não apresentavam a variação de pressão nas três coletas. O comportamento bastante variável da pressão durante a SNN é um padrão observado durante a prática clínica e pode estar relacionado a diversos fatores como estado comportamental, fome, experiência e adaptação do controle motor oral. Outros fatores que podem influenciar nos resultados^(19)^ e que não foram analisados no presente estudo são a idade gestacional e o sexo. Acredita-se ainda que o comportamento de sucção pode ser modificado quando avaliado por meio do dedo enluvado ou protótipo de medição.

#### Pausas

Com relação à variável número de pausas, a segunda coleta apresentou um valor maior, o que é esperado pelo fato de apresentar um número maior de grupos de sucção. Desta forma, observou-se também que o tempo de pausas é significativamente menor na segunda coleta, uma vez que o tempo de sucção se apresentou maior nesta.

O ritmo de sucção é caracterizado pelas eclosões de sucção alternadas com pausas e mostra-se fundamental para a coordenação e eficiência da função no RN^(27)^. Portanto, a análise dos parâmetros número de pausas e tempo de pausas associada ao número de grupos de sucção e tempo dos grupos de sucção torna-se essencial em um instrumento que tem por objetivo fornecer informações quantitativas acerca da SNN.

Vale lembrar que o presente estudo avaliou RNs com estruturas e funções sensório motoras orais íntegras e que algumas alterações podem modificar o padrão de sucção. Como exemplo, uma pesquisa revelou que características anatômicas alteradas do frênulo lingual influenciam o movimento da língua durante a sucção não-nutritiva e o ritmo da sucção durante a amamentação^(28)^.

Autores afirmaram que a duração das pausas entre os grupos de sucção diminui com o aumento da maturação e da atividade de sucção^(29)^. Portanto, a ocorrência e a duração das pausas configuram um parâmetro relevante na avaliação quantitativa da SNN.

#### Sucções esporádicas

Quanto à variável número de sucções esporádicas, observou-se que a segunda coleta apresentou um número significativamente menor quando comparada à primeira e terceira coleta.

Em RNs pré-termos há um padrão desorganizado e com menor número de sucção e que com posterior desenvolvimento da SNN há um aumento da capacidade de modificação do padrão com as experiências apreendidas^(18)^. Assim, embora no presente estudo não tenha sido encontrada diferença, considerando que foram avaliados apenas RN sem alterações, a presença de sucções esporádicas parece ser um parâmetro importante a ser investigado junto a bebês de risco.

### Reprodutibilidade dos parâmetros

Quando realizadas as comparações múltiplas entre os três momentos de coleta, observou-se que as diferenças estatísticas significantes ocorreram entre as coletas um e dois e dois e três. Assim, é possível afirmar que a coleta três não difere da coleta um.

Desta forma, analisando e comparando cada variável separadamente, é possível notar que a segunda coleta apresentou: maior número de grupos de sucção, maior número de sucções, menor tempo para iniciar os grupos de sucção, maior tempo de grupos de sucção, menor número de sucções esporádicas, valores de pressão média maiores e com menor desvio padrão, maior número de pausas com tempo de pausas menor. Esses dados indicam uma maior prontidão para o início do comportamento de sucção, manutenção significativa da pressão média de sucção e do ritmo de sucção.

Considerando-se as comparações sugere-se, portanto, que a primeira medição seja realizada como treinamento, sendo o segundo registro mais confiável para análise do desempenho do RN. Um estudo^(15)^ concluiu que o padrão de sucção é alterado no decorrer das análises, indicando um sinal de aprendizado no início, em que a frequência aumenta gradativamente e que o tempo de sucção sem descanso pode ocasionar diminuição das amplitudes de sucção nas medições finais.

### Análise computacional dos parâmetros

Quanto à análise de concordância apresentada por meio do CCI, foi possível verificar que os dois métodos independentes de avaliação (manual e computadorizado) obtiveram concordância quase perfeita para todos os 12 parâmetros avaliados.

A concordância alta, a garantia de sistematização dos métodos de extração e análise dos dados, a viabilização da aplicação do limiar em todos os registros e o tempo significativamente menor de análise (manual em torno de uma hora por gráfico, *software* em torno de um minuto por gráfico) evidenciam a efetividade do *software* programado no Matlab®. Ademais, outra programação específica realizada no Matlab® já foi descrita como um sistema automático viável para a avaliação objetiva da sucção^(14)^.

Pesquisadores também relataram que ao utilizar métodos independentes de avaliação, dentre eles a identificação visual e um programa de análises de detecção de sucções, as comparações mostraram estreita concordância entre a análise automatizada e a análise manual^(15)^.

A literatura apontou a relevância de métodos diagnósticos para a avaliação dos parâmetros de sucção e da aptidão para alimentação por via oral^(30)^. Também indicou a importância da medição de SNN para o monitoramento das medidas longitudinalmente e seu uso como ferramenta de ensino^(9)^.

A validação de um instrumento de avaliação da sucção não-nutritiva em recém-nascidos influencia positivamente o trabalho dos profissionais que atuam com aleitamento materno, devido a padronização das avaliações e a adequação das condutas terapêuticas.

Como limitações da pesquisa é possível citar o tamanho amostral reduzido e a ausência de avaliação de acordo com o sexo e idade gestacional, uma vez que se tratatou de estudo exploratório inicial. Sugere-se para os próximos estudos, além da análise dessas variáveis: aprimoramento no *design* do aparelho; a avaliação de RNs pré-termo; a avaliação de RNs com alteração do frênulo lingual; análise do comportamento de fadiga ao longo das medições; uso de registro de vídeos durante as avaliações e formulação de protocolo para classificação visual do traçado para que posteriormente possam ser comparadas a avaliação subjetiva e objetiva.

## CONCLUSÃO

As variáveis número de grupos de sucção, número de sucções, tempo para iniciar os grupos de sucção, tempo dos grupos de sucção, frequência de sucção, número de sucções esporádicas, pressão mínima, pressão média, pressão máxima, número de pausas e tempo de pausas mostraram-se relevantes para a avaliação da SNN.

Os dados apontaram que na primeira coleta parece haver um comportamento de treinamento e na segunda observa-se maior prontidão para iniciar a sucção, manutenção da pressão média de sucção e significativo ritmo de sucção.

Desta forma, a utilização do programa em Matlab® para a extração e análise dos parâmetros da SNN mostrou-se viável e eficaz, e a análise pelo programa apresentou alta concordância quando comparada à avaliação manual.
